# Editorial: Pathogens, Pathobionts, and Autoimmunity

**DOI:** 10.3389/fimmu.2021.752980

**Published:** 2021-09-08

**Authors:** Linda A. Spatz, Gregg J. Silverman, Judith A. James

**Affiliations:** ^1^Department of Molecular, Cellular, and Biomedical Sciences, CUNY School of Medicine, The City College of New York, New York, NY, United States; ^2^Departments of Medicine and Pathology, New York University (NYU) Grossman School of Medicine, New York, NY, United States; ^3^Department of Arthritis and Clinical Immunology, Oklahoma Medical Research Foundation, Oklahoma City, OK, United States

**Keywords:** autoimmunity, autoimmune diseases, pathogens, viruses, molecular mimicry, SLE, pathobionts and gut microbiome, dysbiosis

In this Research Topic on Pathogens, Pathobionts and Autoimmunity, we explore how viruses, bacteria, and commensals can contribute to autoimmunity. The articles presented are either reviews of the literature or original data on mechanisms by which pathogens and pathobionts may trigger autoimmune disease. Several articles are devoted to viruses that have previously been associated with autoimmunity. In particular, Epstein Barr virus (EBV) has frequently been implicated in a number of autoimmune diseases and several mechanisms have been identified. These are discussed in the two reviews by Houen et al. (Houen and Trier; Houen et al.) and by Jog and James and in original research articles by Munroe et al. and Farina et al. In their review entitled “Epstein-Barr Virus and Systemic Autoimmune Diseases”, Houen and Trier offer possible mechanistic explanations for how EBV can trigger diverse disease manifestations in different individuals. They suggest that this may be influenced by the predominant cell types that are infected with EBV in different individuals, such as memory B cells or epithelial cells. In their review on “Epstein-Barr virus and Multiple Sclerosis”, Houen et al. focus on recent studies suggesting that EBV-transformed B cells that enter the central nervous system are direct participants in Multiple Sclerosis (MS) pathogenesis and that monoclonal antibody therapy targeting CD20-positive memory B cells is an effective treatment for patients with relapsing remitting MS and with primary progressive MS.

In their review on “Epstein Barr Virus and Autoimmune Responses in Systemic Lupus Erythematosus”, Jog and James discuss several mechanisms by which EBV can contribute to systemic lupus erythematosus (SLE), including molecular mimicry, dysregulated anti-EBV T cell responses, single nucleotide polymorphisms in the CD40, IL10, and CTLA4 genes associated with SLE that also facilitate EBV re-activation, and recently identified SLE risk alleles known to bind strongly to the EBV EBNA-2 protein (1–7). In addition, they describe how EBV viral proteins, such as viral IL-10 (vIL-10) and latent membrane protein 1 (LMP-1), which are homologues of human proteins that alter immune responses, can potentially lead to SLE and how noncoding EBV viral RNAs can lead to activation of IFN-α, a pro-inflammatory cytokine associated with SLE (8, 9). Finally, they summarize several animal models including humanized mice, that are currently being used to study the role of EBV in autoimmunity.

In a primary article in this Research Topic, Munroe et al. examine the role of EBV LMP1 in SLE. LMP1 is a functional mimic of CD40 that can activate B cells. Mice transgenic for a mCD40-LMP1 hybrid molecule have splenomegaly, lymphadenopathy, an expanded population of immature/activated B-lymphocytes and slightly elevated levels of autoantibodies (10). It has previously been shown that mice injected with EBV nuclear antigen 1 (EBNA-1) develop antibodies that cross-react with the ribonucleoprotein, Sm B due to molecular mimicry of a peptide in EBNA-1 that is highly homologous to a peptide in Sm B (11, 12). In the current study, Munroe et al. demonstrate that lymphocytes from mCD40-LMP1 mice injected with EBNA-1 develop significantly greater cellular and humoral immune responses to EBNA-1 and to Sm B than lymphocytes from wild type mice and have elevated ANAs and anti-dsDNA antibodies due to epitope spreading to DNA protein complexes. This suggests that EBV can accelerate autoimmunity in SLE by providing a combination of signals; one that leads to immune dysregulation and another that elicits a cross-reactive autoimmune response.

EBV is also associated with systemic sclerosis (SSc, scleroderma) pathogenesis. In their primary research article, Farina et al. (https://www.ncbi.nlm.nih.gov/pmc/articles/PMC8089375/) demonstrate that EBV can indirectly infect endothelial cells (ECs) using monocytes bound to EBV as a shuttle. They previously demonstrated that monocytes from SSc patients carry EBV and that these monocytes can adhere to the endothelium (13). In the current report, they show that once in ECs, EBV can activate TLR9 and INF-α inducible genes and trigger an innate, pro-inflammatory response that can lead to vascular damage, seen in the early stages of SSc. Understanding the role of EBV in vascular injury in SSc, may lead to therapeutic strategies aimed at preventing viral infection.

In addition to EBV, other viruses have also been associated with autoimmunity. In an extensive review in this Research Topic, Mustelin and Ukadike discuss various mechanisms by which retroviruses and retrotransposons in our genome can contribute to rheumatological autoimmune diseases. A large part of the human genome consists of retroviral sequences or retrotransposons that have been reverse transcribed and integrated into our genome (14). Mustelin and Ukadike cite studies demonstrating that the expression of certain human endogenous retroviral RNAs (HERV-K and HERV-E) and retroviral proteins are increased in SLE and RA patients. These retroviral proteins are believed to elicit autoantibodies. Mustelin and Ukadike also describe studies demonstrating how elevated levels of retrotransposon RNA transcripts such as long interspersed nuclear elements (L1) in SLE patients, can bind the ribonucleoproteins Ro60 and La as well as the viral ORF1p protein to form complexes which can serve as target autoantigens in SLE. In addition, they review how these endogenous viruses can interact with RNA and DNA sensors to elicit the production of IFN-α and thereby promote SLE pathogenesis. Therapeutic strategies that target IFN-α, including the recently FDA-approved anifrolimumab, or that inhibit reverse transcriptase or DNA and RNA sensors, are also discussed.

Many bacteria have also been linked to autoimmune diseases especially those that populate the gut microbiome. Alterations in the types and concentrations of bacteria that normally inhabit the gut microbiome and changes in the environment in the gut have been observed to contribute to autoimmunity. In their review article, Wu et al. discuss how microbial dysbiosis in the gut and disturbances in several pathways that affect the interaction of gut microbes with the host, may play a role in disease pathogenesis in SLE, inflammatory bowel disease (IBD), rheumatoid arthritis (RA), MS, and type I diabetes (https://www.ncbi.nlm.nih.gov/pmc/articles/PMC7786055/). They note how a reduction in anti-inflammatory microbes within the *Lachnospiraceae* family and an increase in pro-inflammatory microbes such as *Ruminococcus gnavus* (that has been reassigned to the genus Blautia) are observed in a subset of patients with IBD, and have been linked to lupus nephritis pathogenesis (15). They also review studies demonstrating how molecular mimicry may be the mechanism by which the gut microbiota is linked to SLE. They describe how autoantibodies and autoreactive T cells specific for Ro60 may arise due to cross-reactivity with a Ro60 ortholog protein produced by gut commensals and how autoantibodies to lipoglycans in *Ruminococcus gnavus* are linked to the generation of anti-dsDNA autoantibodies (15).

In a primary research report, Bagavant et al. (https://pubmed.ncbi.nlm.nih.gov/34122404/), investigate a link between *Enterococcus gallinarum*, a gram-positive bacteria found in the gut, and SLE. They observe that SLE patients with antibodies to Ribosomal P (three phosphorylated proteins on the 60s subunit of ribosomes) have higher titers of IgG antibodies to *E. gallinarum* than healthy individuals. They also observe higher titers of serum IgG antibodies to E. gallinarum in SLE patients with antibodies to dsDNA, Sm, and RNA. They suggest that *E. gallinarum* may influence the subset of autoantigens targeted in lupus patients and infer that molecular mimicry may play a role.

Disruption of the intestinal barrier plays a role in inflammatory autoimmune responses as it allows bacteria to translocate into sites outside the gut that they don’t normally inhabit. In one of two primary research reports in this Research Topic by Zhang et al. they demonstrate that intestinal barrier disruption and translocation of bacteria into the liver can trigger autoimmune hepatitis (AIH). They also show that liver macrophages activated by receptor interacting protein 3 (RIP3), a regulator of necrosis/necroptosis, can contribute to the liver damage observed in AIH by their secretion of pro-inflammatory cytokines. Furthermore, they demonstrate that antibiotic treatment can inhibit RIP3 accumulation and activation of liver macrophages, thereby ameliorating disease. This supports their hypothesis that microbiota play a role in AIH and suggests that RIP3 can be a potential therapeutic target in this autoimmune disease. In their second report in this Research Topic, Zhang et al. examine the role of the commensal gut bacterium *Bifidobacterium animalis* (B420) that is found in probiotics, in the treatment of experimental autoimmune hepatitis in mice (EAH). They observe pathobiont dysbiosis in EAH mice with a reduction in fecal anerobes and an expansion in potentially pathogenic bacteria such as *Bacteroides* and *Ruminococcus*. They also observe that certain short chain fatty acids (SCFAs) such as butyric acids are decreased in AIH patients and EAH mice and that RIP3 is increased. They now demonstrate that B420 treatment can improve the intestinal barrier, increase the level of butyric acids and lower the levels of RIP3, all of which have protective effects. These results reveal that B420 can ameliorate liver damage in EAH mice and suggest that modulation of the gut microbiota with probiotics has potential in the treatment of AIH.

Finally, an original and a review article in this Research Topic examines the role of the gut microbiome in ankylosing spondyloarthropathies. There is much overlap between spondyloarthropathies and gut inflammation, and many patients with a spondyloarthropathy also have gut inflammation, while some patients with IBD have spondyloarthritis (SpA) (16). Therefore, the pathogenesis of these inflammatory diseases may be intertwined. Using metabolomics and metagenomics, Berlinberg et al. address the influence of gut dysbiosis on tryptophan metabolism and the relevance for SpA pathogenesis. They observe that there is an expansion in certain tryptophan metabolites such as indole-3-acetate (IAA) and indole-3-acetaldehyde (I3Ald) in individuals with SpA, irrespective of whether they also have associated Crohn’s disease (CD), suggesting that the presence of these metabolites is specific to SpA. They also observe that the gut communities of SpA patients commonly have increases in the representation of microbial genes involved in tryptophan metabolism, whereas in healthy individuals there is instead an elevation in genes involved in tryptophan synthesis. The authors suggest that the influence of the overall composition of the gut microbiota communities may be more important in the alteration in tryptophan metabolism leading to SpA than individual bacterial species. This hypothesis is supported by Gill and Rosenbaum in their review on “Putative pathobionts in HLA-B27-associated spondyloarthropathy” https://www.ncbi.nlm.nih.gov/pmc/articles/PMC7848169/. They discuss various bacterial, fungal, and viral pathobionts that are associated with HLA-B27 SpAs and overlapping inflammatory diseases of the gut. They observe that different microbes are associated with SpA and CD in different individuals and that there doesn’t appear to be one consensus microbe. They conclude that the microbiome community and the interactions of the gut microbes with one another are what determines whether HLA-B27 individuals will develop inflammatory autoimmunity and that this is influenced by the genetics of the individual and the environment.

In summary, this Research Topic elucidates many different ways that viral pathogens and gut pathobionts can contribute to autoimmunity. Each of these mechanisms may offer individual pathways that can be targeted by therapeutic strategies.

## Author Contributions

There are 3 editors for this Research Topic. Each of the authors contributed equally in inviting contributors for this edition and in reviewing articles submitted for this edition. LAS wrote the editorial with assistance from her co-editors GJS and JAJ. All authors contributed to the article and approved the submitted version.

## Funding

LAS is supported by a grant from the Global Autoimmune Institute (GAI). GJS is supported by NIH grants, P50 AR070591-01A1, R01 AI143313-01U, R01 LM012517, and a grant from the Lupus Research Alliance. JAJ is supported by NIH grants U54GM104938, UM1144292, and P30AR073750.

## Conflict of Interest

The authors declare that the research was conducted in the absence of any commercial or financial relationships that could be construed as a potential conflict of interest.

## Publisher’s Note

All claims expressed in this article are solely those of the authors and do not necessarily represent those of their affiliated organizations, or those of the publisher, the editors and the reviewers. Any product that may be evaluated in this article, or claim that may be made by its manufacturer, is not guaranteed or endorsed by the publisher.
